# Phylosymbiosis shapes skin bacterial communities and pathogen-protective function in Appalachian salamanders

**DOI:** 10.1093/ismejo/wrae104

**Published:** 2024-06-11

**Authors:** Owen G Osborne, Randall R Jiménez, Allison Q Byrne, Brian Gratwicke, Amy Ellison, Carly R Muletz-Wolz

**Affiliations:** School of Environmental and Natural Sciences, Bangor University, Deiniol Road, Bangor, Gwynedd LL57 2DG, United Kingdom; Center for Conservation Genomics, Smithsonian’s National Zoological Park and Conservation Biology Institute, Washington, DC 20008, United States; International Union for Conservation of Nature, C. 39, Los Yoses, San Jose, 146-2150, Costa Rica; Center for Conservation Genomics, Smithsonian’s National Zoological Park and Conservation Biology Institute, Washington, DC 20008, United States; Department of Environmental Science, Policy and Management, University of California, Berkeley, CA 94720-3114, United States; Center for Species Survival, Smithsonian’s National Zoological Park and Conservation Biology Institute, Front Royal, VA 22630, United States; School of Environmental and Natural Sciences, Bangor University, Deiniol Road, Bangor, Gwynedd LL57 2DG, United Kingdom; Center for Conservation Genomics, Smithsonian’s National Zoological Park and Conservation Biology Institute, Washington, DC 20008, United States

**Keywords:** phylosymbiosis, Batrachochytrium dendrobatidis, Batrachochytrium salamandrivorans, host-microbiome interactions, community assembly, probiotics

## Abstract

Phylosymbiosis is an association between host-associated microbiome composition and host phylogeny. This pattern can arise via the evolution of host traits, habitat preferences, diets, and the co-diversification of hosts and microbes. Understanding the drivers of phylosymbiosis is vital for modelling disease-microbiome interactions and manipulating microbiomes in multi-host systems. This study quantifies phylosymbiosis in Appalachian salamander skin in the context of infection by the fungal pathogen *Batrachochytrium dendrobatidis* (Bd), while accounting for environmental microbiome exposure. We sampled ten salamander species representing >150M years of divergence, assessed their Bd infection status, and analysed their skin and environmental microbiomes. Our results reveal a significant signal of phylosymbiosis, whereas the local environmental pool of microbes, climate, geography, and Bd infection load had a smaller impact. Host-microbe co-speciation was not evident, indicating that the effect stems from the evolution of host traits influencing microbiome assembly. Bd infection is correlated with host phylogeny and the abundance of Bd-inhibitory bacterial strains, suggesting that the long-term evolutionary dynamics between salamander hosts and their skin microbiomes affect the present-day distribution of the pathogen, along with habitat-linked exposure risk. Five Bd-inhibitory bacterial strains showed unusual generalism: occurring in most host species and habitats. These generalist strains may enhance the likelihood of probiotic manipulations colonising and persisting on hosts. Our results underscore the substantial influence of host-microbiome eco-evolutionary dynamics on environmental health and disease outcomes.

## Introduction

**Figure 1:**
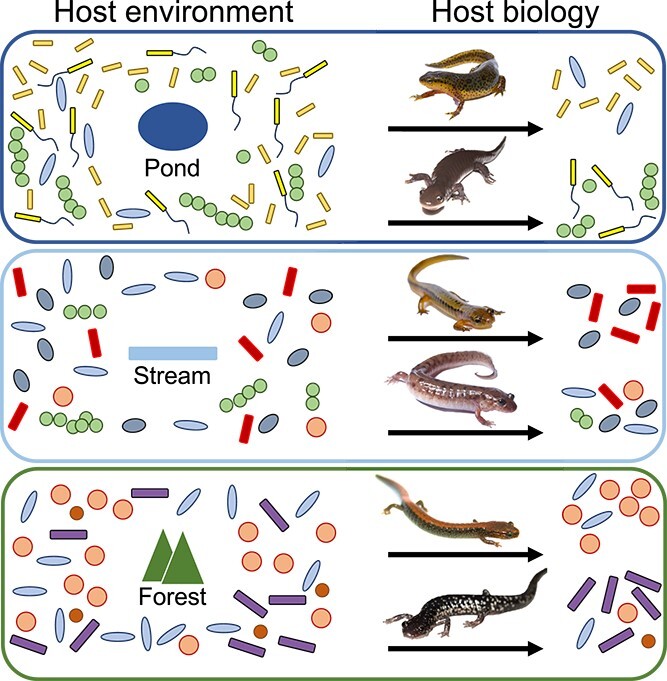
Conceptual diagram illustrating how host environment impacts the regional pool of microbes, whereas species-specific host traits filter this pool of microbes.

Host-associated microbiomes are ecosystems structured by a combination of deterministic and stochastic processes [1–5]. Compared to other complex multispecies assemblages, host-associated microbiomes are unique in that assembly processes act at both host environment and host biology levels [6]. The environment of the host often affects the regional pool of microbial species that exist as potential colonisers [7]. In host-associated microbiomes, microbes colonise a living organism, and a secondary ecological filter operates at the host biology level (Fig. 1). This could relate to the host species or host site, such as a plant root or an animal skin [8]. Host filtering may involve traits that are evolutionarily conserved or subject to divergent selection between host species. Using an integrated approach to examine environmental and host microbiomes in evolutionary diverse host species communities will allow us to more accurately quantify the processes that underpin host-associated microbiome assembly.

In host-associated microbiome research, individuals within the same species have most often been sampled in multiple localities as a proxy for different environmental exposures. Generally, the environment has been found to influence microbiomes in a variety of plant [9, 10] and animal systems, including skin microbiomes [11, 12]. Although these studies have been powerful in demonstrating the role of the environment, few studies have characterised the microbiome of the host’s environment in parallel with that of the host microbiome. Integration of environmental microbiomes with host microbiomes provides critical insight into the role of environmental transmission of microbiota from environment to host [7], which can impact host ecology [13] and health [14].

Host biology is often important in predicting microbial composition and microbiomes typically differ between species [4, 5, 11]. These differences sometimes mirror host evolutionary history—a pattern termed phylosymbiosis—whereby microbiome dissimilarity is correlated with host phylogenetic distance [15, 16]. Phylosymbiosis has been observed in vertebrate gut [17, 18] and skin microbiomes [19–21], internal plant microbiomes [22], and many other systems [16]. Host-microbe co-speciation accounts for phylosymbiosis in some cases [23]. However, mechanisms of ecological filtering by host traits can mirror evolutionary history and explain phylosymbiosis [24], such as co-variation between host diet or life history and phylogeny [25, 26]. Although phylosymbiosis has been demonstrated in multiple systems, the mechanisms underlying it, and particularly the influence of host life history, are poorly understood.

The eco-evolutionary processes shaping host-associated microbiomes may have significant practical implications for biodiversity conservation. The emerging field of wildlife probiotics [27] has the potential to effectively mitigate wildlife outbreaks, and probiotics have been applied to multiple animal diseases, including white-nose syndrome in bats [28], chytridiomycosis in amphibians [29], and American foulbrood in honeybees [30]. For these interventions to be effective, however, some degree of persistence of the introduced probiotics is required, and this is likely to be largely dependent on the host-microbiome interactions that play out in wild settings. Therefore, understanding the dynamics of host-associated microbiome specificity and host-microbe co-evolution is crucial for designing effective microbiome-manipulation strategies to combat pathogen-mediated biodiversity loss.

Among vertebrates, amphibian skin is an important system to examine environmental and host evolutionary effects on microbiome assembly [31]. Amphibian skin lacks protective fur or feathers and is covered with a moist mucus layer, which can act as a bacterial substrate [31]. Furthermore, in contrast to other vertebrate classes, amphibian skin is a critical respiratory and osmoregulatory organ [31]. It also plays an important role in innate immunity, hosting an extremely diverse array of antimicrobial peptides [32]. Thus, amphibians are particularly sensitive to skin microbiome perturbations but are equipped with unique adaptations to influence their skin microbiome composition.

The Appalachian Mountains are rich in salamander species diversity, with >75 species in 14 genera. These are dominated by members of the family *Plethodontidae* but represent >150M years of evolution in total [33, 34]. These species also differ widely in life histories, ranging from fully aquatic to fully terrestrial species, with many species co-occurring. Further, amphibians are impacted by the chytrid fungal pathogens (*Batrachochytrium dendrobatidis* [Bd] and *Batrachochytrium salamandrivorans* [Bsal]), which can infect their skin and cause the disease chytridiomycosis [35]. Not all amphibian species are equally susceptible, and skin microbiomes play an important role in Bd infection probability and disease outcomes [4, 36]. Together, the high species diversity, range of environmental exposures, and pathogen-protective traits of the skin microbiome make Appalachian salamanders a useful study system to examine the effects of environment, life history, pathogen susceptibility, and evolutionary history on host microbiome assembly.

Here, we studied the environmental and host skin-associated bacteria from 10 wild salamander species in the Central Appalachians, USA. Specifically, we aimed to: (i) investigate the roles of geographic locality, habitat, and host species in salamander-associated microbial community structure; (ii) determine whether, and by what mechanism, skin microbiomes follow a pattern of phylosymbiosis in salamanders; (iii) determine whether these host-microbiome eco-evolutionary processes affect disease dynamics via Bd-protective bacteria in wild salamanders; and (iv) explore whether this information can be used to develop more effective pathogen mitigation strategies. Integrating evolutionary history and environmental microbiomes into a unified framework allows us to identify how these combined factors impact host-associated microbiomes and organismal and environmental health.

## Methods

### Sample collection

**Figure 2:**
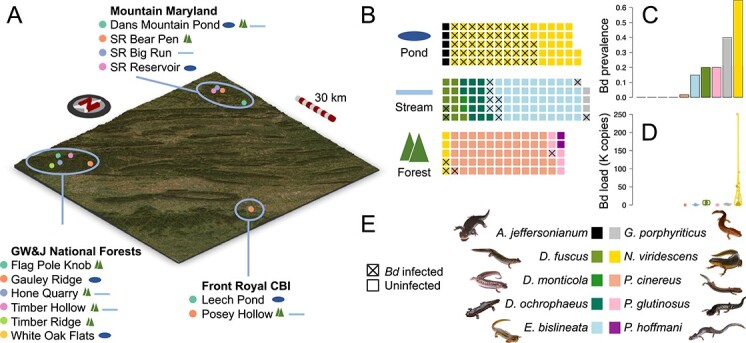
Sampling scheme. Sampling sites within each of the three localities: Mountain Maryland, George Washington and Jefferson National Forests and Front Royal Conservation Biology Institute, are shown on a topological map (A). Sites are distinguished by point colour within each locality, and habitat types are shown as icons (as in panel B) to the right of each site name. Waffle plots (B) show the number of individuals of each species in each habitat type. Each square represents one individual, coloured by species as in panel E. *Batrachochytrium dendrobatidis* (Bd) infected individuals are indicated by a cross. Bd prevalence is shown as a bar plot (panel C; proportion of infected individuals) and Bd load is shown as a violin plot (panel D; units of 1000 copies; jittered points show actual values for all infected individuals). Bars and violins are coloured by species as in E. Photographs in E are all the authors’ work except for *P. hoffmani*, which is available on a creative commons licence (CC-BY-NC, © Josh Emms 2018).

We sampled 10 species of salamander at 12 sites within three localities in Maryland and Virginia, USA, in October 2020 (permit details: Supplementary Methods 1). This included species: *Ambystoma jeffersonianum* (5 samples; 1 site), *Desmognathus fuscus* (11 samples; 3 sites), *D. monticola* (2 samples; 1 site), *D. ochrophaeus* (13 samples; 2 sites), *Eurycea bislineata* (53 samples; 5 sites), *Gyrinophilus porphyriticus* (5 samples; 2 sites), *Notophthalmus viridescens* (77 samples; 6 sites), *Plethodon cinereus* (57 samples; 5 sites), *P. glutinosus* (6 samples; 2 sites), and *P. hoffmani* (2 samples; 2 sites; Fig. 2; Tables S1 and S2). Salamanders and their environment were sampled from one or more of three broad habitats at each site: pond, stream, or forest (Table S1). Salamanders were captured by dip-netting (ponds) and visual encounter surveys by flipping logs and rocks (streams and forests) at each of the sites. Each captured salamander was swabbed for disease quantification and microbiome profiling before being released. Environmental samples from aquatic and terrestrial environments were collected from substrates (water for aquatic samples or soil for terrestrial samples) near where salamanders were captured (Supplementary Methods 2).

### Pathogen and microbiome molecular methods

Genomic DNA was extracted from skin swabs using the DNeasy PowerSoil HTP 96 kit (Qiagen). We used qPCR for the quantification of Bd, Bsal, and ranavirus infection using synthesised gene fragments (gBlocks; Integrated DNA) as in [37] and reported Bd loads as Bd copies per swab. Based on previous studies, loads above 10 000 copies were considered high and suggestive of a diseased state [38, 39]. All swabs were tested in duplicate. We used a two-step PCR library preparation and dual-index paired-end sequencing to sequence the skin microbiome of each salamander skin swab sample, as well as positive and negative controls. Briefly, we amplified the V3–V5 region of the 16S rRNA gene (~380 bp) using the universal primers 515F-Y and 939R [4, 5], and sequenced the libraries on two MiSeq (Illumina) runs at the Center for Conservation Genomics, NZCBI (Supplementary Methods 3).

### Sequence processing

Raw data processing followed a previous study [4], using the *dada2* [40], *MAFFT* [41], *FastTree* [42], *QIIME 2* [43], *phyloseq* [44], and *decontam* [45] software packages and taxonomic identification using the Ribosomal Database Project [46] database (Supplementary Methods 4). Our sequencing produced a total of 12 217 181 sequences with an average of 34 031 read pairs per sample (Table S2). A rarefied dataset was created by rarefying at an even depth of 2945 reads, which was chosen to capture the diversity present while retaining as many samples as possible (Fig. S1). This was used to account for uneven sampling depths in some downstream analyses (noted below). All ASV sequences were BLASTn searched against the Antifungal Isolates Database [47] (updated database received from M. Bletz, July 2022). ASVs with 100% identity to known Bd-inhibitory isolates from the database were considered to have putative Bd-inhibitory activity.

### Microbial diversity analyses

We estimated alpha diversity (ASV richness) using the rarefied dataset. To determine whether alpha-diversity significantly differed according to locality, habitat, and host species, we used Scheirer–Ray–Hare tests [48] (SRH tests; used due to inequality of variance between groups) implemented in the R package “rcompanion” [49]. To circumvent the confounding effect of species and habitat in salamander samples, we only compared species within the same habitat category in habitat subsets (Supplementary Methods 5). One species was found in both pond and forest habitats as adults, *N. viridescens*, and was analysed as a species subset to examine locality and habitat effects within a species (Supplementary Methods 5). For all significant factors with more than two levels in the SRH tests, we conducted *post-hoc* Dunn’s tests implemented in the R package “FSA” [50] to determine which groups significantly differed (Supplementary Methods 5). We also estimated the alpha diversity of Bd-inhibitory ASVs and correlated Bd-inhibitory ASV richness with total ASV richness using a linear model (LM).

We estimated beta diversity (from the rarefied dataset) between all sample pairs using the Jaccard, Bray–Curtis, unweighted UniFrac, and weighted UniFrac metrics. We then used PermanovaG tests implemented in the “GUniFrac” R package [51], which allowed all four beta-diversity metrics to be combined in a single omnibus test, to test differences in bacterial community composition associated with locality, habitat, and host species (with species examined within habitat-specific subsets as in alpha diversity; Supplementary Methods 5). For significant *PermanovaG* tests, we conducted *post-hoc* testing using pairwise *PermanovaG* to determine which groups significantly differed (with *P* values corrected using FDR). Community composition differences were visualised using Nonmetric Multidimensional Scaling (NMDS).

### Phylosymbiosis analyses

To determine whether microbial community distance showed a signal of phylosymbiosis, we used both Mantel test and tree-based methods. We also tested for an association between mean univariate microbial traits (ASV richness, Bd-inhibitory bacterial richness and relative abundance, Bd prevalence, and Bd load) using the function “multiPhylosignal” in the R package “picante” [52, 53]. To determine whether the phylogenetic signal in these traits was robust to intraspecific variability, we used a bootstrapping approach. For each bootstrap replicate, we resampled each species with replacement maintaining the original number of samples per species, recalculated each mean trait, and re-ran the “multiPhylosignal” test. This was repeated 1000 times, and *P-*values for all replicates were combined using the Cauchy combination method [54]. For phylosymbiosis analyses, we implemented all tests using each of the four beta-diversity measures of salamander skin calculated above separately. We first extracted a dated phylogeny for all host species from TimeTree [55] (downloaded: 31 July 2023; TimeTree synthesises multiple published phylogenies, in this case 16) and extracted host phylogenetic distance using the “cophenetic” function in the R package “ape” [56]. Mantel tests were conducted at both the sample and species level, and a tree-based permutation test was implemented with the “cospeciation” function in the R package “phytools” [57] (Supplementary Methods 6).

To quantify the effect of host phylogeny and environmental variables on the salamander skin microbiome, we used multiple regression on distance matrices [58, 59] (MRM) implemented in the “MRM” function in the R package “ecodist” [60]. Our MRM model included five predictor variables: host phylogenetic distance, geographic distance, climatic distance, environmental microbiome distance, and Bd infection load distance, which were standardised, so the analysis resulted in comparable standardised regression coefficients (*β*; Supplementary Methods 7). To determine whether Bd-inhibitory strains showed a similar pattern of phylosymbiosis to the general microbiome, phylosymbiosis and MRM analyses were repeated using only the Bd-inhibitory subset of taxa.

The species are geographically and habitat-restricted, and some bacterial taxa may only be present in certain habitats or geographic regions. Phylosymbiosis could therefore plausibly result from habitat and range differences coinciding between host and bacterial species. We addressed this possibility through two approaches. First, to test whether differential presence-absence of bacteria between localities and habitats drives phylosymbiosis, we produced a “global-ASVs” dataset by filtering the salamander microbiome dataset to include only ASVs, which were present in all locality-habitat combinations (in either environmental or skin salamander skin samples in the pre-rarefied data; 66 ASVs). All phylosymbiosis tests and MRM analyses were then repeated with this dataset. Second, to determine whether phylosymbiosis was evident when the influence of habitat was removed, we repeated the individual-level Mantel tests and MRM analysis on subsets containing only species from stream and forest habitats separately (pond species were not used in this analysis because only two were sampled).

One mechanism that may lead to phylosymbiosis is the co-speciation of hosts and vertically acquired microbes during diversification [61]. To determine whether this process contributes to phylosymbiosis in the salamander skin microbiome, we used the ParaFit [62] method (Supplementary Methods 8).

### Specificity analysis

To quantify the specificity of ASVs in the salamander skin microbiome to host phylogeny, environment, and Bd load, we used the method implemented in the “specificity” R package [63]. This approach indicates whether ASVs occupy a narrower (or broader) range of an environmental variable than expected by chance using the convenient Rao’s quadradic entropy [64, 65], which allows calculation of specificity for both linear and higher-dimensional variables. We calculated specificity to the five explanatory variables used in the MRM analysis. Using the rarefied dataset, ASVs occurring in fewer than 10 samples were removed (as recommended by the authors). We calculated specificity indices and *P-*values for all remaining ASVs and calculated mean specificity indices for all branches of the bacterial phylogeny, which were visualised with heat trees implemented in the R package “metacoder” [66]. Specificity indices were examined for putative Bd-inhibitory ASVs to identify strains that may have good potential to become established when introduced to novel host environments and thus be promising candidates for probiotic approaches to control Bd.

### Relationship between Bd infection and microbiome structure

To identify ASVs that significantly differed between Bd-infected and non-infected individuals, we conducted differential abundance analysis implemented in the “DESeq2” R package [67, 68]. This was conducted at the ASV level using the model formula “~Species_Habitat + Locality + Bd_infection_,” where Species_Habitat is a combined factor of Species and Habitat, Locality is geographic locality, and Bd_infection_ indicates whether salamanders were infected or not. Corrected *P*-values were then extracted for the Bd-infected versus non-infected contrast. To account for the high sparsity of the ASV level data, we used the “poscounts” method for estimating size factors (used to correct for different library sizes between samples). We cross-referenced all ASVs to the putatively anti-Bd ASV set. We determined whether these were significantly over-represented among significant ASVs using a Fisher’s exact test.

## Results

### Microbial diversity is associated with geography, habitat, and host species

**Figure 3:**
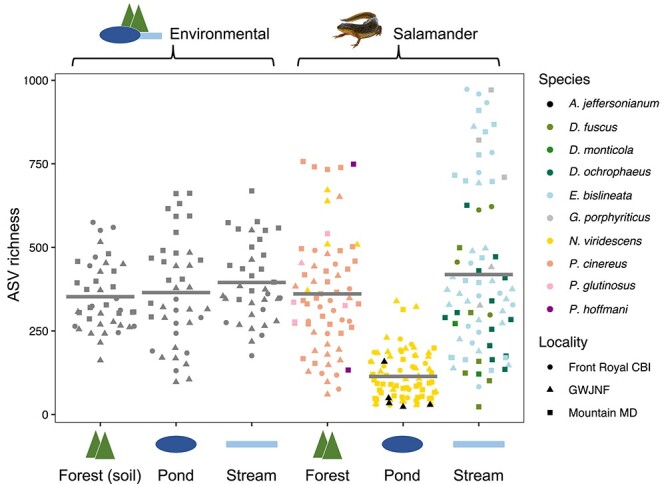
Alpha diversity for all samples. Beeswarm plots show ASV richness for all environmental (left) and salamander skin samples (right), grouped by habitat on the *x*-axis. Each point represents a single sample and points with similar ASV richness values are separated on the *X*-axis to minimise overlap. Horizontal bars show the mean for each habitat. Point colour indicates host species and shape indicates locality.

**Figure 4:**
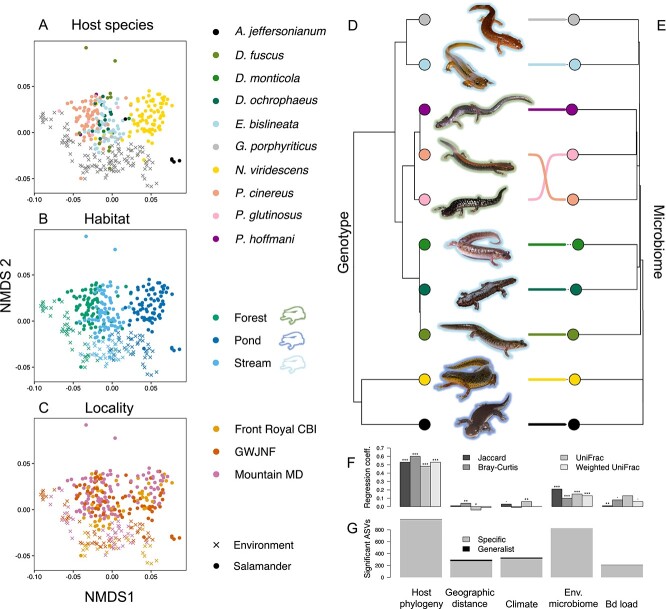
There is a strong signal of phylosymbiosis in Appalachian salamander skin microbiomes. NMDS plots based on Bray-Curtis distance (A–C) for all salamander skin and environmental samples. Each point represents a single sample and points are coloured by host species (A), habitat (B), or locality (C). Point shapes show sample type (i.e. salamander skin or environmental samples). A dated host phylogeny (D) is shown beside neighbour-joining based hierarchical clustering of mean pairwise Bray-Curtis microbiome distance between each salamander species pair (E). Coloured lines link the same species between the two dendrograms. Coloured tip points indicate species, and outlines around salamander images indicate primary habitat. Results of multiple regression on distance matrices (MRM) analysis (F) show the effect of multiple explanatory variables of skin microbiome distance. Bar plots show standardised regression coefficients for host phylogeny, geographic distance, climate distance, environmental microbiome distance, and Bd load using four different skin-microbiome beta-diversity statistics. Stars above each bars indicate significance (*P* < .001: ^*^^*^^*^; *P* < .01: ^*^^*^; *P* < .05: ^*^). In our specificity analysis (G), there were most significantly specific ASVs for host phylogeny, followed by environmental microbiome distance.

After filtering ASVs and removing low read-count and control samples, 118 environmental and 222 salamander samples remained with between 23 and 973 ASVs (Table S2). We first assessed the effects of locality, habitat, and species on environmental and skin microbiome structure. Generally, environmental microbiome structure (Figs 3 and 4A–C) differed among localities (alpha and beta diversity) and habitats (beta diversity), and salamander skin microbiome structure (Figs 3 and 4A–C) differed among localities (beta diversity), habitats (alpha and beta diversity), and salamander species (alpha and beta diversity; Tables S3–S6). Salamanders living in ponds had markedly lower bacterial diversity on their skin, but this was not observed in environmental samples (Fig. 3; Table S3). One species, *N. viridescens*, was present in two habitats and showed lower alpha diversity and distinct bacterial composition (beta diversity) in ponds as aquatic adults compared to terrestrial adults in the forest (Table S3). For environmental samples, Mountain Maryland environments had significantly higher alpha diversity than the other two localities (Table S4), whereas bacterial community composition significantly differed among all localities and habitats (Fig. 4A–C; Fig. S2; Tables S5 and S6). Bd-inhibitory bacterial richness was correlated with total ASV richness on salamander skin and in the environment (LM: *P* < .001), but the relationship was stronger on salamander skin (*R*^2^ skin = 0.50, environment = 0.11). For salamander samples, bacterial community composition significantly differed among all three localities (Tables S5 and S6) and species in all three habitat subsets (Table S5). Eight species pairs from a total of 17 tested were significantly different in the post-hoc tests (Table S6). Except for *D. fuscus* and *D. ochrophaeus*, all significant pairs were from different genera, implying that greater host phylogenetic distance may be associated with higher microbiome divergence, a hypothesis that we then tested explicitly.

### Skin microbiome distance recapitulates host phylogeny

We found a strong pattern of phylosymbiosis in the skin microbiome of Appalachian salamanders (Fig. 4D–E). We likewise found a phylogenetic signal in bacterial ASV richness, Bd-inhibitory bacterial richness, and Bd load and a near-significant effect in Bd prevalence, all of which remained significant when we accounted for intraspecific variation (Table S7). In our phylosymbiosis analyses, individual-level Mantel tests were significant (*P* < .0001) for all four beta-diversity metrics. Species-level Mantel tests were all significant, apart from weighted UniFrac (*P* = .0511; Table S8). Tree-based permutation tests were consistently significant (Table S8). We obtained similar results when using the subset of Bd-inhibitory ASVs (Table S8). When we calculated beta-diversity statistics using only the set of ASVs present in all habitat-locality combinations (66 global ASVs), the pattern of phylosymbiosis remained (Table S8). Similarly, a significant signal of phylosymbiosis was found (Table S8), and host phylogeny had a significant effect (albeit with a reduced effect size; Table S9) within single-habitat subsets. These results indicated that the signal of phylosymbiosis is not derived solely from differences in bacterial presence or absence between the habitats and ranges of the salamander species. The results of the MRM analysis supported this conclusion. Host phylogeny showed a strong (*β =* [0.48, 0.60]; Fig. 4f; Fig. S3; Table S9) and highly significant (*P* < .0001) association with skin microbiome dissimilarity across all beta-diversity metrics. Environmental microbiome distance was also significant for all beta-diversity metrics (*P* < .0001) but had a smaller effect size (*β =* [0.10, 0.21]; Fig. 4f; Table S9). The results for climatic, geographic, and Bd load distance were more inconsistent, with significant effects using some beta diversity metrics but with consistently small effect sizes (Fig. 4f; Table S8). For the subset of Bd-inhibitory ASVs, host phylogeny again showed the strongest association with skin microbiome dissimilarity (*β* = [0.35, 0.52], *P* < 0.0001). Putatively, Bd-inhibitory ASVs were consistently associated with Bd load (*β* = [0.07, 0.19]), in contrast to the complete dataset (Fig. S3; Table S9).

We found no evidence that vertical transmission was driving phylosymbiosis in Appalachian salamander skin. Across all four clustering methods, no OTU had a significant phylogenetic signal following multiple test correction (Table S10).

### Host and environmental specificity

**Figure 5:**
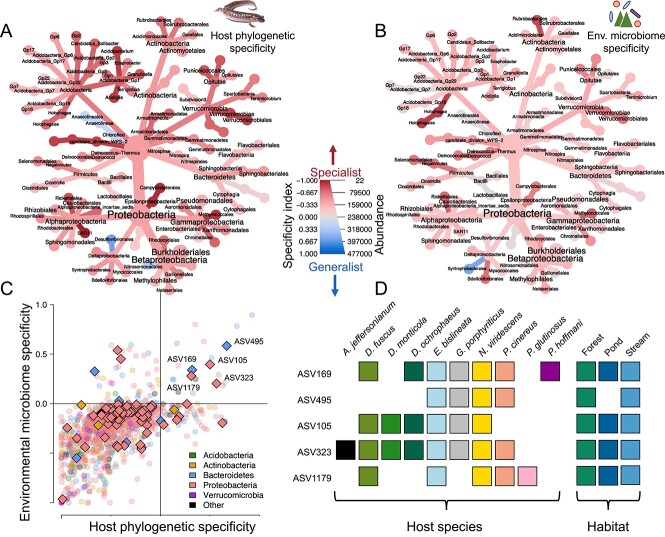
Host and environmental specificity across the salamander skin microbiome and in known anti-Bd taxa. The bacterial phylogeny of the skin microbiome is shown to order level, with colour indicating mean specificity index to host phylogeny (A) and environmental microbiome distance (B). A specificity index <0 indicates higher specificity and specificity index >0 indicates higher generalism. The dot plot (C) shows the relationship between these metrics with each point representing a single ASV, coloured by phylum. Known anti-Bd ASVs are shown as diamonds and all other ASVs are shown as circles. Known anti-Bd taxa with the lowest specificity (i.e. most positive specificity index) are present in a wide range of host species and habitats (D). Coloured squares indicate presence in each host species and habitat for the most generalist anti-Bd ASVs.

Specificity to host phylogeny, climate distance, geographic distance, environmental microbiome distance, and Bd load varied between microbial phyla (Fig. 5A and B; Figs S5–S7). The highest number of significantly specific ASVs were found for host phylogeny, followed by environmental microbiome distance (Fig. 4G; Table S11). There were substantially fewer geography- and climate-specific ASVs, in line with the MRM analysis (Fig. 4F). Of the putative anti-Bd bacteria, five had positive (i.e. generalist) specificity indices for both host and environmental microbiome distances (Fig. 5C; Table S11). These were taxonomically identified as *Chryseobacterium* sp. (ASV169, 495), *Iodobacter* sp. (ASV105), *Acinetobacter* sp. (ASV323), and an ASV of an unknown genus in the family *Enterobacteriaceae* (ASV1179). The final two of these also had positive indices for both climate and geographic distance (correlation between the different specificity indices was high; Fig. S4), and all were present in a wide range of species and habitats (Fig. 5D). These five taxa may be particularly good candidates for probiotic treatments (e.g. [14]) due to their ability to colonise a broad range of salamander species and environments in the wild.

### Known Bd-inhibitory bacteria are significantly associated with Bd infection status in wild salamanders

Bd was found to be widespread in the Appalachian region we sampled, with prevalence ranging from 0% to 65% per species (Fig. 2). Six of the ten salamander species were found to be infected, with *N. viridescens* having the highest prevalence and loads (prevalence: 65%; mean Bd load: 17 317 copies), and *G. porphyriticus*, *P. glutinosus*, *D. fuscus*, *E. bislineata*, and *P. cinereus* also found to have at least one individual infected (Fig. 2). No individuals were infected with Bsal or ranavirus. We identified 547 taxa that were differentially abundant between Bd-infected and non-infected individuals while controlling for species, habitat, and locality (Table S11; negative log fold change represents lower abundance in Bd-infected salamanders). These were dominated by *Proteobacteria* (247 ASVs), but also included taxa distributed widely across the bacterial phylogeny, including *Bacteroidetes* (125 ASVs), *Actinobacteria* (80 ASVs), *Acidobacteria* (46 ASVs), and *Verrucomicrobia* (17 ASVs; Table S11). Although many bacteria have been shown to have Bd-inhibitory ability *in vitro* [47], these are largely untested in the wild. We therefore cross-referenced the differentially abundant taxa and those with significant specificity to Bd load (see above) with the putatively anti-Bd set of ASVs identified *in vitro* from a previously published database [47]. This revealed that, whereas only 3% of ASVs absent from the Bd-inhibitory database were differentially abundant according to Bd infection status, 16% of putatively anti-Bd bacteria were, a highly significant association (Table S12). A similar significant association was found between Bd load specificity and putative anti-Bd activity (Table S12). Of the 22 putative anti-Bd bacteria significantly associated with Bd infection status, 13 ASVs were more abundant in Bd-infected individuals, whereas nine ASVs were more abundant in uninfected individuals. When these were considered separately, the relationship with putative anti-Bd activity remained significant (*P* < .0001; Table S12).

## Discussion

Microbial diversity plays a crucial role in the health of humans and many animals and plants [69–71]. Determining which factors impact host-associated microbiome structure is critical for issues as diverse as health, food production, and biodiversity conservation [72, 73]. Here, we show that in the world’s foremost centre of salamander biodiversity, microbiome composition follows a pattern of phylosymbiosis and is influenced by habitat, and that the abundance of many bacterial taxa is linked to pathogen (Bd) infection in the wild. Together, this indicates that salamander skin microbiomes are constrained in their functional capacity by the evolutionary history and environment of the host, and that these eco-evolutionary processes may alter disease dynamics by affecting the distribution of pathogen-protective bacteria.

### Phylosymbiosis in Appalachian salamanders is not explained by habitat and range divergence

We provide strong evidence for phylosymbiosis in the skin microbiomes of Appalachian salamanders in terms of overall bacterial composition, putatively Bd-inhibitory bacterial composition, and widely distributed bacterial composition (global-ASVs). Host phylogeny consistently explained bacterial composition more strongly than the environment or the local pool of microbes (Fig. 4F; Table S9). Phylosymbiosis refers to a general pattern where relationships between host-associated microbial communities recapitulate the host phylogeny. The term does not imply a mechanism and may arise via either stochastic processes, such as ecological drift and isolation-by-distance, or deterministic processes [61]. These can include the evolution of host traits and environmental preferences that affect their microbial community compositions or co-diversification between host and microbes. Simulations have shown that weighted measures detect phylosymbiosis more effectively, potentially because bacteria that are better adapted to the host environment are likely to be more abundant [24]. We observed greater support for phylosymbiosis in weighted beta diversity measures in the MRM analyses and in Mantel tests for the putatively Bd-inhibitory and global-ASVs datasets, supporting this hypothesis (although not in the Mantel tests of the whole microbiome dataset).

Ecological filtering from the environment may explain patterns of phylosymbiosis [74], but here we account for environmental microbiome contribution and find a far stronger effect from host phylogeny, suggesting a greater role of the evolution of intrinsic host traits on microbiome assembly. A recent study showed phylosymbiosis in salamander skin microbiomes [21], but it did not quantify the contribution of environmental microbial community differences due to the absence of environmental microbiome samples. Phylosymbiosis in salamanders contrasts with a previous study in Malagasy frog skin microbiomes, where host ecology was found to be a more important driver than host phylogeny [75]. Drivers of rapid diversification may explain this difference. In Malagasy frogs [76], morphological and microhabitat-niche linked diversification predominate. In Appalachian salamanders, climatic-niche diversification predominates, and morphology and microhabitat use are generally not linked to diversification [77]. Appalachian salamanders may have undergone diversification in host skin traits in response to exposure to climatic-specific environmental microbes. However, we found no evidence for co-speciation between salamander species and microbial symbionts, a result that could be confirmed in future work using metagenomic approaches with greater phylogenetic resolution [61]. This suggests instead that Appalachian salamanders are predominately acquiring their skin microbial symbionts from the environment during an individual’s lifetime. Nonetheless, we found that the environmental pool of microbes had a relatively small effect on the skin microbiome. Taken together, we hypothesise that the evolution of host climatic or habitat preference results in a different pool of microbes that could colonise the host skin, but that the evolution of intrinsic host traits, potentially immunological traits, such as antimicrobial peptides (AMPs) [4] and major histocompatibility complex (MHC) molecules [78], has a more important effect by differentially filtering these microbes between species (Figs 1 and 4F).

Host-microbe skin interactions are particularly important in relation to skin-associated pathogens, such as Bd. We found large differences in Bd prevalence and load between species, with Bd load having a significant phylogenetic signal. Species-specific differences in Bd prevalence and load may result from intrinsic host traits, such as MHC genes and AMPs [32, 79], and the differences in skin-associated microbiota as documented here and previously [4, 21], but may also relate to the linkage between evolutionary history and habitat preference in the salamander species we examined. We hypothesise that both evolutionary history and habitat-linked exposure risk explains Bd load dynamics, and not habitat alone. This is supported by: (i) high Bd susceptibility and Bd-linked decline in terrestrial Plethodontid salamanders [80, 81] and (ii) our observations that Bd infected individuals occurred in all habitats and localities.

We show evidence for evolutionary history predicting the distribution of Bd-inhibitory bacterial richness and composition. We also identified 547 ASVs that were significantly differentially abundant between *Bd-*infected and non-infected individuals, suggesting that skin microbiome-Bd dynamics are also operating at more recent time scales*.* Strains with known Bd-inhibitory activity *in vitro* were significantly more likely to be differentially abundant between Bd-infected and uninfected salamanders. These included strains that were both significantly more and less abundant in infected versus uninfected individuals, hinting at a diversity of mechanisms by which Bd-protective bacteria may benefit hosts in the wild. It is possible that some strains may preclude Bd infection entirely, whereas others may reduce the severity of symptoms in infected individuals. Of the 13 genera of differentially abundant anti-Bd bacteria, only three genera had multiple ASVs that were differentially abundant—*Chryseobacterium*, *Pedobacter*, and *Pseudomonas*—and these genera had ASVs that showed both increased and decreased abundance with Bd infection, highlighting the importance of strain level distinctions. Another finding was the low bacterial richness in pond-dwelling salamanders, particularly *N. viridescens*, which also have high Bd prevalence and harbour very high Bd loads. Our results highlight the important links among evolutionary history, environmental and host microbial diversity, and disease dynamics [4].

### Host-specificity and *in vivo* effectiveness of putative anti-Bd probiotics

The use of probiotics to improve host health has been applied in fields as diverse as aquaculture [82], crop improvement [83], human health [84], and conservation biology [85]. This includes efforts to harness bacteria with antifungal activity to combat chytridiomycosis in amphibians [86, 87]. This approach may have several advantages over other approaches. For example, the long-term establishment of protective bacteria would provide lasting protection without the need for repeated treatments as required with antifungal chemical agents, and the use of bacteria already occurring in the ecosystem would reduce the chance of damaging and unpredictable ecosystem impacts [88]. However, although many bacterial strains have been shown to have anti-Bd activity *in vitro* [47], application of these to ameliorate Bd infection in live amphibians has had mixed results [85, 87, 89]. Low colonisation and persistence of anti-Bd bacteria on the host may limit effectiveness [14, 87, 90]. We identify bacteria in our specificity analyses that are more generalist and likely have a greater probability of persisting when introduced to novel hosts or environments (16S sequences available in Table S11). These may improve the chance of effective anti-Bd activity in wild populations and may be particularly relevant if the Bd sister taxon, Bsal, were to reach Appalachia, as we have previously shown that Bd-inhibitory bacteria can also kill Bsal [91]. Taxa that are known to have generalist distributions (Fig. 5C) may be fruitful targets for future probiotic approaches, specifically *Chryseobacterium* and *Acinetobacter*, which have already been isolated from multiple continents and diverse species [47] and shown to be important in pathogen-microbiome-host interactions [92, 93]. Probiotic effectiveness using multiple probiotic strains challenged against diverse clades of Bd and Bsal should be considered in picking ideal strains to focus research efforts on [91]. Conversely, taxa from more species-specific clades, such as *Acidobacteria* and *Actinobacteria*, may have a lower chance of being effective as general anti-*Bd* probiotics.

Here, we show that microbial diversity in Appalachian salamander skin shows a strong pattern of phylosymbiosis, which likely derives largely from the evolution of intrinsic host traits that select for unique microbial symbionts from the environmental pool. Furthermore, the abundance of multiple microbial taxa is significantly associated with fungal Bd infection, including strains that are known to have anti-Bd activity *in vitro*. Our results highlight the importance of the long-term evolutionary dynamics of host-microbiome interactions in disease susceptibility and suggest potential avenues by which to harness them more effectively in conservation interventions. Deepening our understanding of the complex interactions between pathogen, microbiome, environment, and host immune system that determine disease susceptibility increases our chances of improving disease outcomes for conservation purposes, both for Bd and to combat Bsal, should this sister taxon be introduced into this salamander biodiversity hotspot.

## Supplementary Material

Osborne_supplementary_final_clean

Osborne_Supplementary_tables_R1_wrae104

## Data Availability

We deposited demultiplexed sequence data in the National Center for Biotechnology Information Sequence (NCBI) under BioProject ID: PRJNA1039858. All code used for data analysis is available at https://github.com/ogosborne/salamander_phylosymbiosis and all software versions are shown in Table S13.
