# Supplementary material for: Phylosymbiosis shapes skin bacterial communities and pathogen-protective function in Appalachian salamanders
Source: ISME J. 2024 Jun 11;18(1):wrae104. doi: 10.1093/ismejo/wrae104 (PMC11195472; doi:10.1093/ismejo/wrae104)
Supplement: Osborne_supplementary_final_clean [file osborne_supplementary_final_clean.pdf]

Supplementary materials: Phylosymbiosis shapes skin bacterial communities and pathogen-protective function in Appalachian salamanders

Owen G. Osborne, Randall R. Jiménez, Allison Q. Byrne, Brian Gratwicke, Amy Ellison, Carly R. Muletz-Wolz

Supplementary methods.

Supplementary methods 1. Sampling permissions.

We had IACUC approval from NZCBI (#18-19) and scientific collection permits issued from the US Fish and Wildlife Service (MA93679B), Virginia Department of Wildlife Resources (067393), and Maryland Department of Natural Resources (DNR; 57633) as well as permission from Maryland DNR to sample in State Parks (2020DNR259) and a permit waiver from US Forest Service to sample in GWJNF.

Supplementary methods 2. Sample collection.

Salamanders and their environment were sampled from one of three broad habitats at each site: pond, stream, or forest (Table S1). Salamanders were captured by dip-netting (ponds) and visual encounter surveys by flipping logs and rocks (streams and forest) at each of the sites. Each salamander was rinsed with sterile Nanopure water (Barnstead International, IA) and then placed in individual sterile Whirl-Pak bags (Nasco, WI). Each captured salamander was swabbed 25 times (dorsal/ventral sides, front/back limbs, tail; five strokes each) for disease quantification and microbiome profiling, before being released. Environmental samples from aquatic and terrestrial environments were collected from substrate near where salamanders were captured. To collect aquatic samples (ponds and streams), we moved a sterile swab through water for 15s at a depth of ~20 cm. To collect terrestrial forest samples, we moved a sterile swab through the soil at a depth of ~2 cm for 15 s, respectively. Swabs were placed in sterile 2 mL tubes, placed on dry ice for transport and stored at -20°C until extraction. *N. viridescens* was the only species to occur in two habitats (forest and pond). While a terrestrial juvenile stage exists in this species (efts), all individuals sampled were adults. Individuals in the forest habitat were terrestrial adults that leave ponds in winter in some areas of their range to overwinter in the forest.

Supplementary methods 3. Pathogen and microbiome molecular methods.

Genomic DNA was extracted from skin swabs using the DNeasy PowerSoil HTP 96 kit (Qiagen 12955) following manufacture's protocol except a 90 sec bead beating step was used on a high-throughput cell disrupter (Mini BeadBeater 1001, Biospec). Positive (Zymo, Catalog No. D6300) and negative extraction controls were extracted with each set of swab extractions.

We used qPCR for the detection of Bd, Bsal and ranavirus infection following [1] and using TaqMan Fast Advanced master mix (ThermoFisher, Cat No. 4444556). All swabs were tested in duplicate. For pathogen quantification, we created Gblock

standards [1] and ran them in duplicate at concentrations of 10^5, 10^4, 10^3, 10^2, 10 and 1 copies with each set of reactions along with duplicate PCR negative controls. We considered a sample positive if it amplified at least twice and if the sample amplified before the 1 copy standard.

We used a two-step PCR library prep and dual-index paired-end Illumina sequencing to sequence the skin microbiome of each salamander skin swab sample, positive controls (ZymoBIOM- ICS microbial community standards; Zymo Catalog No. D6300 & D6305) and negative controls (extraction and PCR negative controls). Briefly, we amplified the V3-V5 region of the 16S rRNA gene (~380 bp) using the universal primers 515F-Y and 939R[2, 3], and sequenced the libraries on two Illumina MiSeq runs (v3 chemistry: 2 × 300 bp kit) at the Center for Conservation Genomics, NZCBI. We deposited demultiplexed Illumina sequence data in the National Center for Biotechnology Information Sequence (NCBI) under BioProject ID: PRJNA1039858.

Supplementary methods 4. Sequence processing.

The *dada2* [4] pipeline was used to quality filter raw reads (maxEE = 2; truncLen = 270[R1] ,180[R2]; trimLeft = 19; trimRight = 23; default for all other parameters), process them into amplicon sequence variants (ASVs), remove chimeras, and assign bacterial taxonomy (using the Ribosomal Database Project [5] trainset 16 [release 11.5]). ASV sequences were aligned and used to produce a phylogenetic tree using the *MAFFT* [6] and *FastTree* [7] algorithms in *QIIME 2* [8]. ASV tables, phylogeny, taxonomy and metadata were then imported into *phyloseq* [9] for further filtering and downstream analysis. Singletons, ASVs with less than 10 total reads and those classified as mitochondria, Cyanobacteria/chloroplast, Archaea, or without a phylum-level classification were removed. The *decontam* [10] R package was used to further remove potential contaminants based on their presence in negative control samples (method = “prevalence”, threshold = 0.1).

Supplementary methods 5. Alpha diversity tests.

All environmental samples were included in one SRH test with locality and habitat as independent variables. For salamander skin samples, 9/10 species only occurred in a single habitat. Therefore, we conducted separate SRH tests for each habitat with host species and locality as explanatory variables. *Notophthalmus viridescens* was the one species found in both forest and pond environments. Therefore, we tested the effect of habitat and locality as explanatory variables for only *N. viridescens* samples on alpha diversity. For all significant factors with more than two levels in the SRH tests, we conducted post-hoc Dunn’s tests implemented in the R package FSA [11] to determine which groups significantly differed. Dunn test *P*-values were corrected for multiple testing using the false discovery rate (FDR) method [12].

Supplementary methods 6. Phylosymbiosis tests.

Mantel tests were implemented using the function *mantel* in the R package *vegan* [13]. We applied two Mantel test approaches: first, we tested distance matrices comparing all salamander skin samples. Second, since concerns have been raised about the possibility of zero-branch lengths biasing Mantel tests containing multiple

conspecifics [14], we implemented a second approach using median microbiome distance for each species pair. For the tree-based approach, we produced a hierarchical clustering of salamander species' microbiomes using a neighbour joining approach implemented in the *nj* function in the R package *ape* [15] based on median distance between salamander skin samples. We tested whether this was significantly more similar to the host phylogeny than expected by chance by using a permutation test implemented in the *cospeciation* function in the R package *phytools* [16] (using 10,000 permutations and Robinson-Foulds).

Supplementary methods 7. Environmental distance calculations and MRM.

Geographic distance was calculated as the Vincenty distance using the function *geodist* in the R package *geodist* [17]. To calculate climatic distance, we extracted the 19 bioclimatic variables for each sampling site from the WorldClim dataset [18] (0.5 minute resolution). These were combined with elevation, and used to perform a principle component analysis using the *dudi.pca* function in the R package *ade4* [19]. Pairwise climatic distance was computed as the Euclidean distance between the scores of the first four principle components, following [20]. Since the environment is the ultimate source of the salamander skin-associated microbiome, we derived a measure of environmental microbiome distance from our environmental samples. This was calculated as the mean Bray-Curtis distance between each locality-habitat combination in the environmental samples. To calculate Bd load distance, we first calculated log-transformed Bd load as $\log_{10}(ZGE + 1)$. The logarithm was used to ensure that infected individuals with differing Bd loads had more similar values to each other than to uninfected individuals. Log-transformed Bd load values were then used to calculate pairwise distances between all sample pairs. All predictor variables were standardised and MRM was conducted with 10,000 permutations and Spearman's correlation coefficient. The test was run separately with the four beta-diversity metrics.

Supplementary methods 8. ParaFit Analysis.

Co-diversification between host and microbes can only be detected in microbial clades which have diverged over the same timescale as the host species. An appropriate clustering threshold to separate such bacterial clades is difficult to estimate, so we clustered ASVs into operational taxonomic units (OTUs) using four different methods [21]: with the *VSEARCH* [22] *cluster_size* algorithm at 95%, 97%, and 99% similarity, and using *Swarm* [23], which does not use a global similarity threshold. We then ran the ParaFit algorithm on each OTU. The ParaFit algorithm requires a host phylogeny, a bacterial phylogeny and a matrix of interactions (i.e. presence/absence) between each host-ASV pair. To produce this input data, we first merged samples of the same species and rarefied the species-merged dataset to an even depth. For each OTU, we then subset the bacterial phylogeny to include only ASVs clustered into the focal OTU. For OTUs which contained at least three ASVs, and for which three host species had one or more OTUs, we ran the ParaFit analysis using the *parafit* function in *ape* with 10,000 permutations and Cailliez correction. We then adjusted P-values to account for multiple testing using FDR.

Supplementary references

1. Standish I, Leis E, Schmitz N, Credico J, Erickson S, Bailey J, et al. Optimizing, validating, and field testing a multiplex qPCR for the detection of amphibian pathogens. *Dis Aquat Organ* 2018; **129**: 1–13.
2. Jiménez RR, Carfagno A, Linhoff L, Gratwicke B, Woodhams DC, Chafran LS, et al. Inhibitory bacterial diversity and mucosome function differentiate susceptibility of Appalachian salamanders to chytrid fungal infection. *Appl Environ Microbiol* 2022; **88**: e01818-21.
3. Keady MM, Jimenez RR, Bragg M, Wagner JCP, Bornbusch SL, Power ML, et al. Ecoevolutionary processes structure milk microbiomes across the mammalian tree of life. *Proc Natl Acad Sci* 2023; **120**: e2218900120.
4. Callahan BJ, McMurdie PJ, Rosen MJ, Han AW, Johnson AJA, Holmes SP. DADA2: High-resolution sample inference from Illumina amplicon data. *Nat Methods* 2016; **13**: 581–583.
5. Maidak BL, Olsen GJ, Larsen N, Overbeek R, McCaughey MJ, Woese CR. The Ribosomal Database Project (RDP). *Nucleic Acids Res* 1996; **24**: 82–85.
6. Katoh K, Misawa K, Kuma K, Miyata T. MAFFT: a novel method for rapid multiple sequence alignment based on fast Fourier transform. *Nucleic Acids Res* 2002; **30**: 3059–3066.
7. Price MN, Dehal PS, Arkin AP. FastTree 2 - Approximately maximum-likelihood trees for large alignments. *PLoS One* 2010; **5**: e9490.
8. Bolyen E, Rideout JR, Dillon MR, Bokulich NA, Abnet CC, Al-Ghalith GA, et al. Reproducible, interactive, scalable and extensible microbiome data science using QIIME 2. *Nat Biotechnol* 2019; **37**: 852–857.
9. McMurdie PJ, Holmes S. Phyloseq: An R package for reproducible interactive analysis and graphics of microbiome census data. *PLoS One* 2013; **8**: e61217.
10. Davis NM, Proctor DM, Holmes SP, Relman DA, Callahan BJ. Simple statistical identification and removal of contaminant sequences in marker-gene and metagenomics data. *Microbiome* 2018; **6**: 226.
11. Ogle DH, Doll JC, Wheeler AP, Dinno A. FSA: simple fisheries stock assessment methods. R package version 0.9.4. 2023.
12. Benjamini Y, Hochberg Y. Controlling the false discovery rate: a practical and powerful approach to multiple testing. *J R Stat Soc Ser B* 1995; **57**: 289–300.
13. Oksanen J, Blanchet FG, Kindt R, Legendre P, O'Hara RB, Simpson GL, et al. vegan: community ecology package; R package. 2010.
14. Song SJ, Sanders JG, Delsuc F, Metcalf J, Amato K, Taylor MW, et al. Comparative analyses of vertebrate gut microbiomes reveal convergence between birds and bats. *MBio* 2020; **11**: e02901-19.
15. Paradis E, Claude J, Strimmer K. APE: Analyses of phylogenetics and evolution in R language. *Bioinformatics* 2004; **20**: 289–290.
16. Revell LJ. phytools: An R package for phylogenetic comparative biology (and other things). *Methods Ecol Evol* 2012; **3**: 217–223.
17. Padgham M. geodist: Fast, dependency-free geodesic distance calculations. R package version 0.0.7. 2021.
18. Fick SE, Hijmans RJ. WorldClim 2: new 1-km spatial resolution climate surfaces for global land areas. *Int J Climatol* 2017; **37**: 4302–4315.

19. Dray S, Dufour A-B. The ade4 package: Implementing the duality diagram for ecologists. *J Stat Softw* 2007; **22**: 1–20.
20. Ramírez-Barahona S, González-Serrano F, Martínez-Ugalde E, Soto-Pozos A, Parra-Olea G. Host phylogeny and environment shape the diversity of the salamander skin microbiome. *Anim Microbiome* 2023; **5**: 52.
21. Perez-Lamarque B, Morlon H. Comparing different computational approaches for detecting long-term vertical transmission in host-associated microbiota. *Mol Ecol* 2023; **32**: 6671–6685.
22. Rognes T, Flouri T, Nichols B, Quince C, Mahé F. VSEARCH: A versatile open source tool for metagenomics. *PeerJ* 2016; **4**: e2584.
23. Mahé F, Rognes T, Quince C, de Vargas C, Dunthorn M. Swarm: Robust and fast clustering method for amplicon-based studies. *PeerJ* 2014; **2**: e593.

Supplementary Figures.

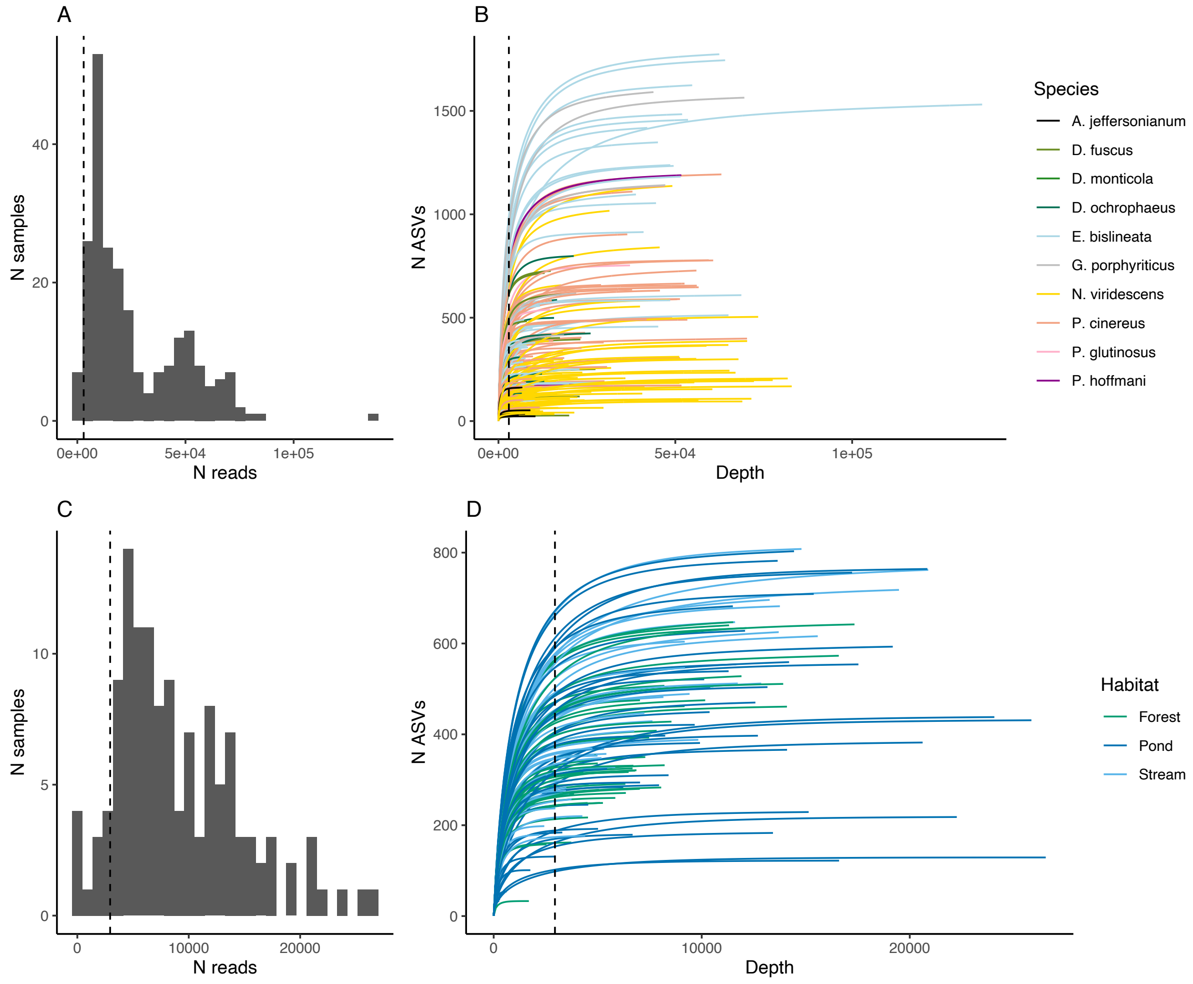

Fig. S1. Sample depth and rarefaction. Histograms show the distribution of number of read pairs per sample for salamander (A) and environmental (C) samples. Rarefaction curves for each sample (step size = 100) are shown for salamander (B) and environmental (D) samples. Dashed vertical lines on all panels show the rarefaction depth used.

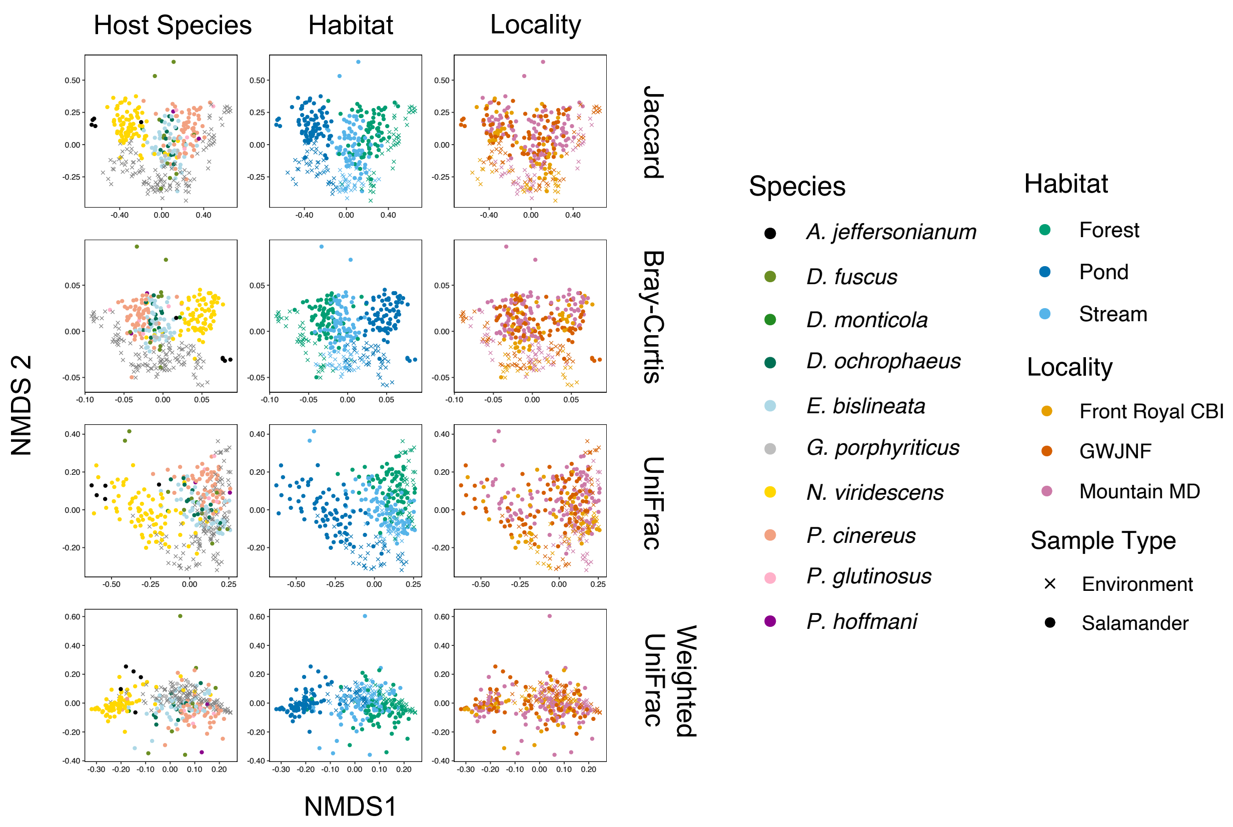

Fig. S2. Non-metric Multidimensional Scaling (NMDS) analysis of all samples. Each row shows the analysis for a different beta-diversity statistic: Jaccard, Bray-Curtis, UniFrac, or Weighted UniFrac. Within each row, each column shows the same plot coloured by species, habitat, or geographic locality.

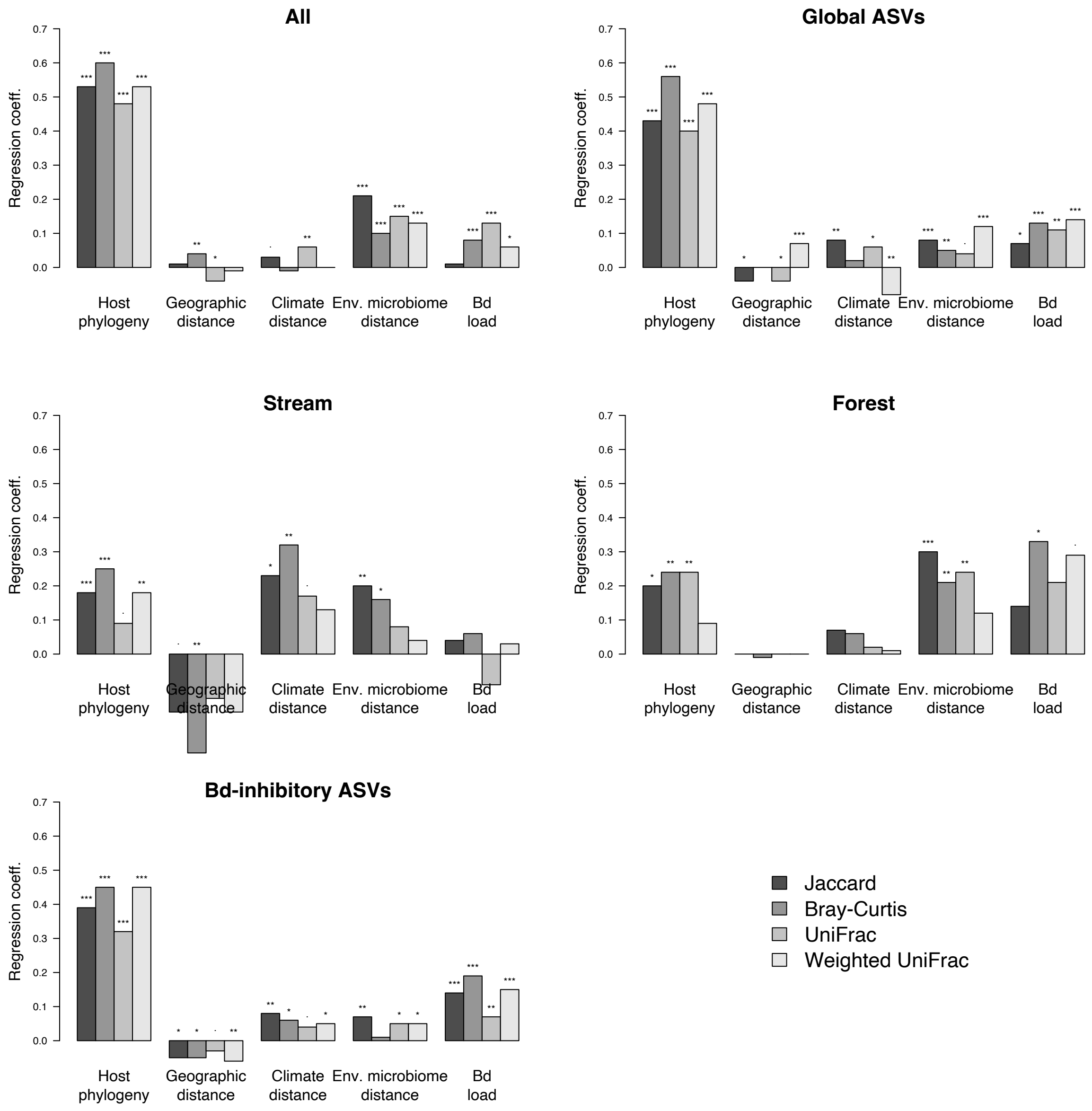

Fig. S3. Results of multiple regression on distance matrices (MRM) analysis using the whole salamander dataset (All; as in fig. 4), only ASVs present in all habitat-locality combinations (Global ASVs), only stream salamander samples (Stream), only forest salamander samples (Forest), and only putatively Bd-inhibitory ASVs (Bd-inhibitory ASVs). Bar plots show standardised regression coefficients for host phylogeny, geographic distance, climate distance, environmental microbiome distance and log10(Bd load + 1) using four different skin-microbiome beta-diversity statistics. Stars above each bars indicate significance (P < 0.001: ***; P < 0.01: **; P < 0.05: *; P < 0.1: .).

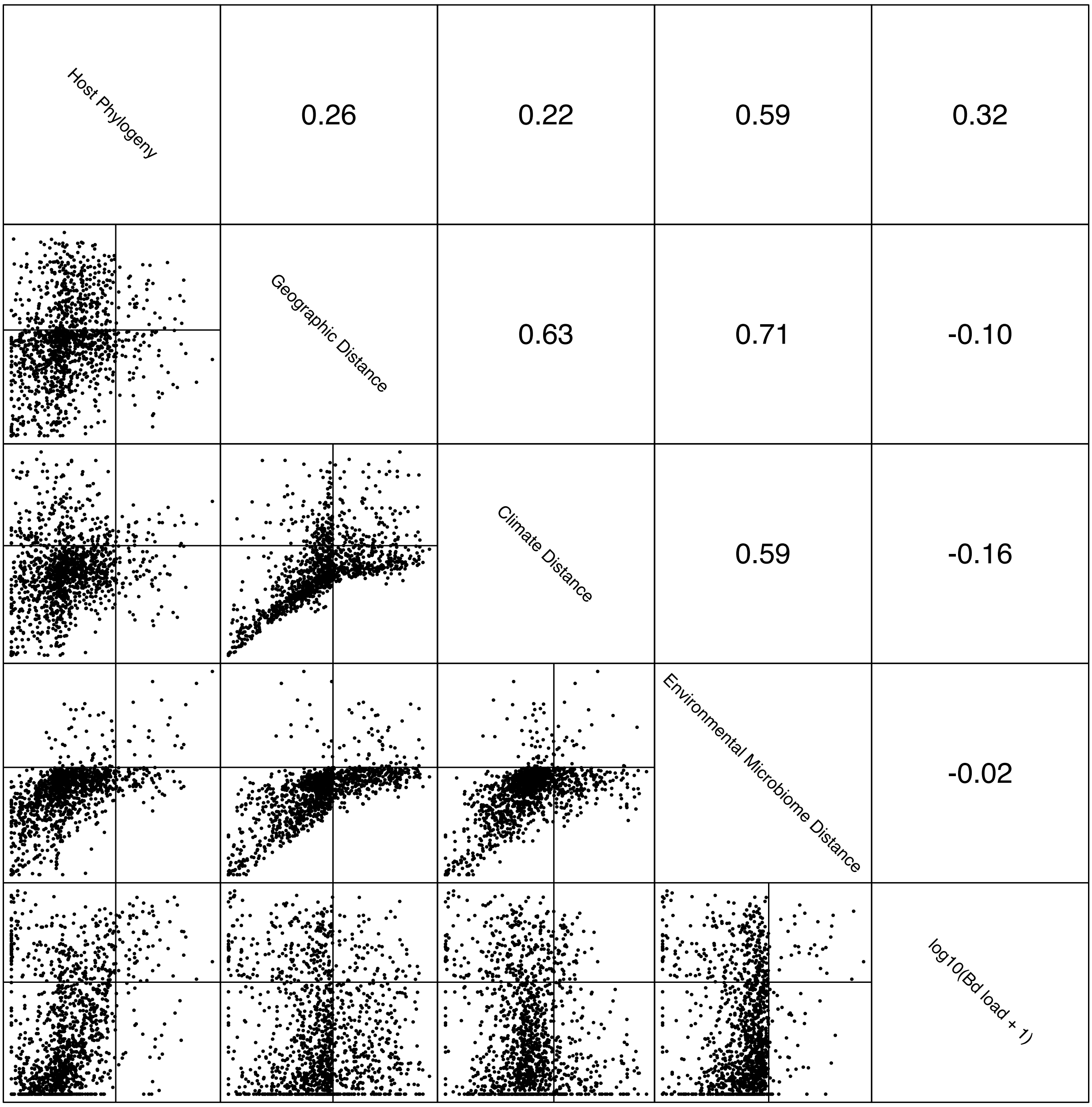

Fig. S4. Correlations between specificity indices. The lower triangle shows dot plots comparing each specificity index, with each point representing a single ASV. The upper triangle contains Pearson correlation coefficients for each pairwise comparison.

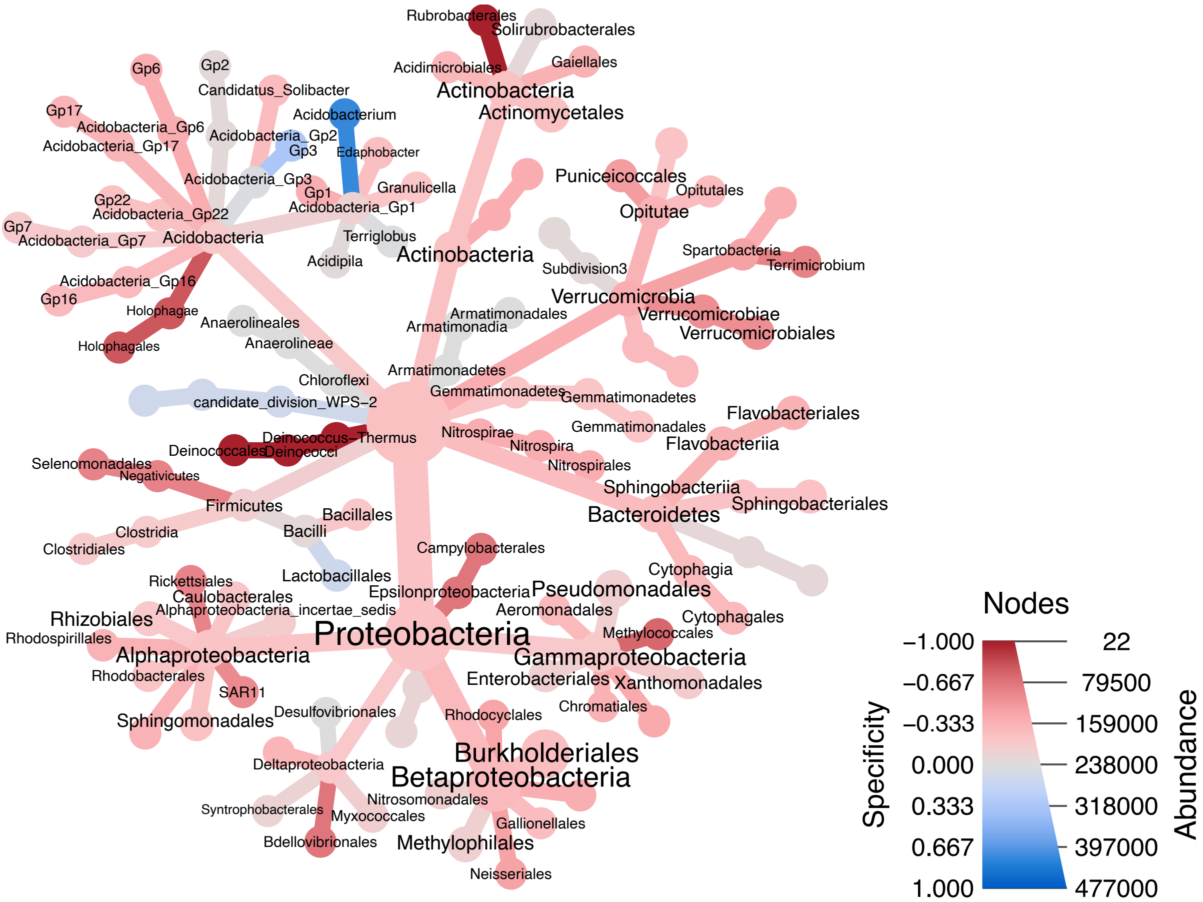

Fig. S5. Heat tree showing mean specificity index to climate distance.

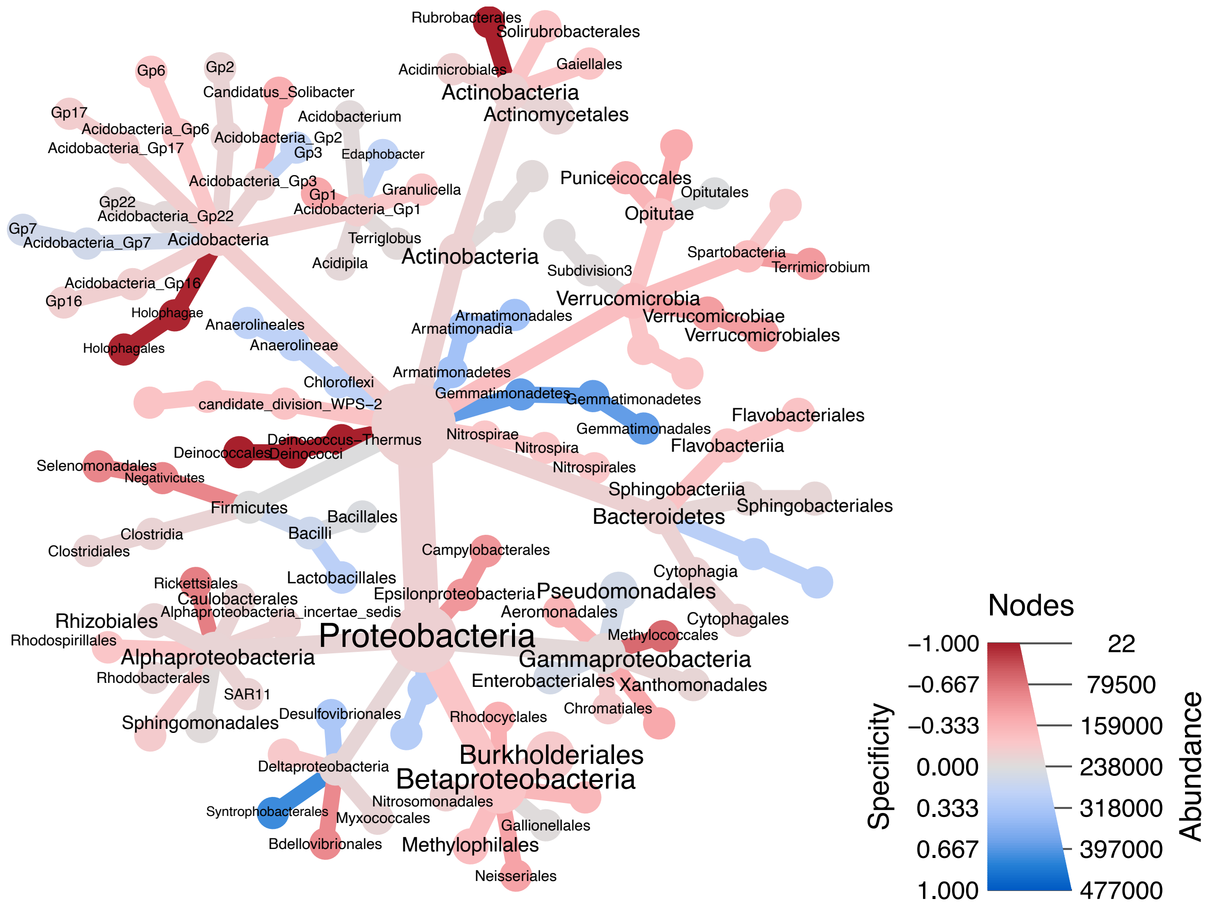

Fig. S6. Heat tree showing mean specificity index to geographic distance.

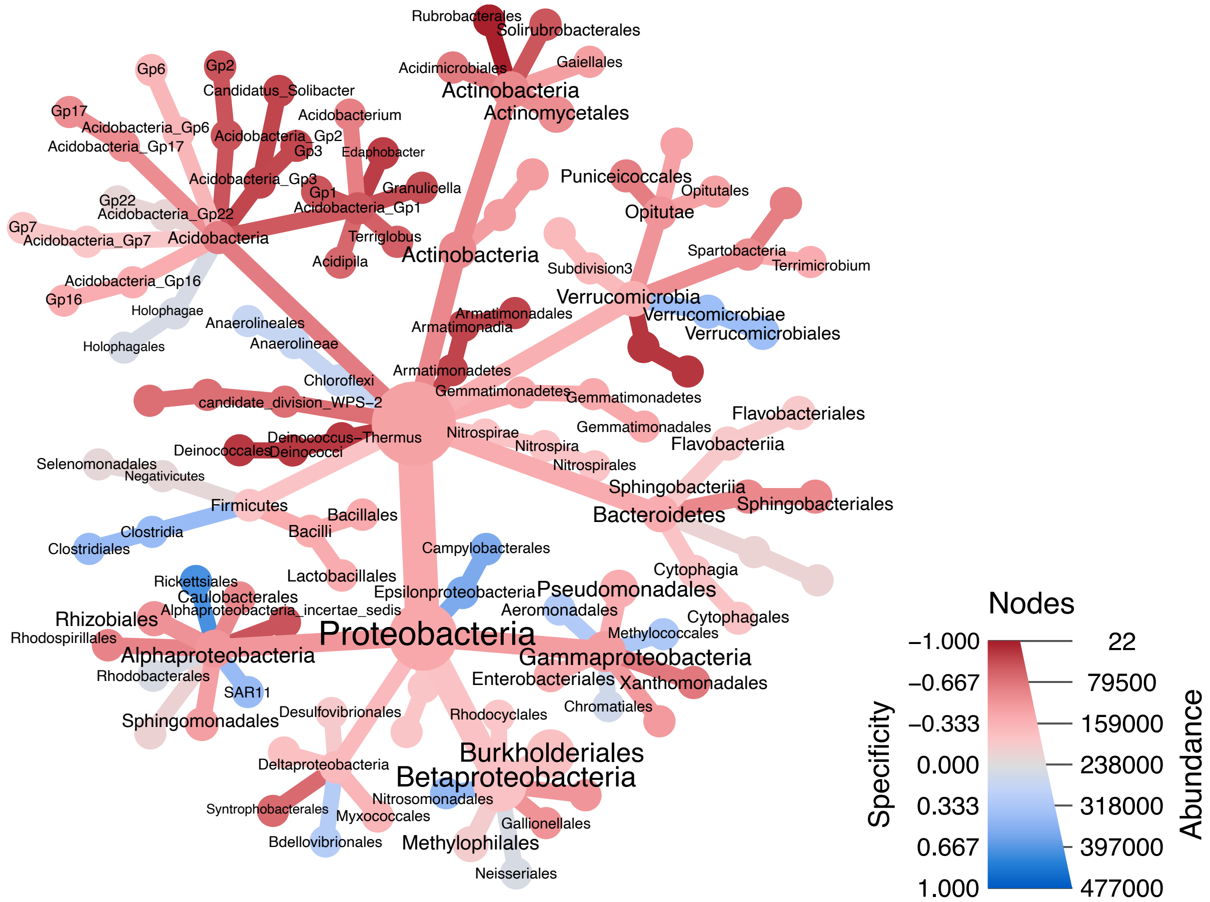

Fig. S7. Heat tree showing mean specificity index to Bd load.
